# Criticality of neuronal avalanches in human sleep and their relationship with sleep macro- and micro-architecture

**DOI:** 10.1016/j.isci.2023.107840

**Published:** 2023-09-09

**Authors:** Silvia Scarpetta, Niccolò Morisi, Carlotta Mutti, Nicoletta Azzi, Irene Trippi, Rosario Ciliento, Ilenia Apicella, Giovanni Messuti, Marianna Angiolelli, Fabrizio Lombardi, Liborio Parrino, Anna Elisabetta Vaudano

**Affiliations:** 1 Department of Physics, University of Salerno, 84084 Fisciano, Italy; 2 INFN sez. Napoli Gr. Coll. Salerno, 84084 Fisciano, Italy; 3 Nephrology, Dialysis and Transplant Unit, University Hospital of Modena, 41121 Modena, Italy; 4 Sleep Disorders Center, Department of Medicine and Surgery, University of Parma, 43121 Parma, Italy; 5 Department of Neurology, University of Wisconsin, Madison, WI 53705, USA; 6 Department of Physics, University of Naples “Federico II”, 80126 Napoli, Italy; 7 Engineering Department, University Campus Bio-Medico of Rome, 00128 Roma, Italy; 8 Institute of Science and Technology Austria, Am Campus 1, 3400 Klosterneuburg, Austria; 9 Department of Biomedical Sciences, University of Padova, Via Ugo Bassi 58B, 35131 Padova, Italy; 10 Neurology Unit, Azienda Ospedaliero-Universitaria of Modena, OCB Hospital, 41125 Modena, Italy; 11 Department of Biomedical, Metabolic and Neural Sciences, University of Modena and Reggio Emilia, 41125 Modena, Italy

**Keywords:** Biological sciences, Neuroscience

## Abstract

Sleep plays a key role in preserving brain function, keeping brain networks in a state that ensures optimal computation. Empirical evidence indicates that this state is consistent with criticality, where scale-free neuronal avalanches emerge. However, the connection between sleep architecture and brain tuning to criticality remains poorly understood. Here, we characterize the critical behavior of avalanches and study their relationship with sleep macro- and micro-architectures, in particular, the cyclic alternating pattern (CAP). We show that avalanches exhibit robust scaling behaviors, with exponents obeying scaling relations consistent with the mean-field directed percolation universality class. We demonstrate that avalanche dynamics is modulated by the NREM-REM cycles and that, within NREM sleep, avalanche occurrence correlates with CAP activation phases—indicating a potential link between CAP and brain tuning to criticality. The results open new perspectives on the collective dynamics underlying CAP function, and on the relationship between sleep architecture, avalanches, and self-organization to criticality.

## Highlights

• During sleep avalanches follow scaling laws in line with a critical branching process
• The measured critical exponents satisfy theoretically predicted scaling relations
• NREM-REM sleep cycle modulates avalanche dynamics
• Activation phases of cyclic alternating patterns correlate with avalanches occurrence

## Graphical abstract

**Figure:**
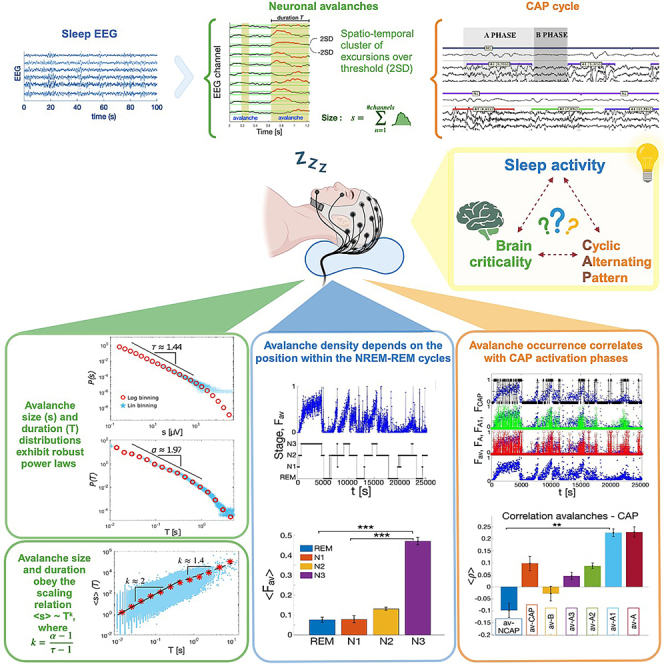

## Introduction

Sleep is an active and dynamic complex process regulated by mechanisms that guide the alternation of non-rapid eye movement (NREM) and rapid eye movement (REM) sleep across the night. Physiologically, sleep macro-architecture is characterized by the concentration of deep slow wave sleep (SWS) (stage N3) in the first half of the night, and the dominance of light sleep (mainly N2) and REM sleep in the second half of the night, a balanced skewness modulated by the homeostatic process and by the REM-off and REM-on systems.^1^ Throughout the night, numerous transitions among these sleep stages occur, and, within sleep stages, brain activity fluctuates in amplitude and frequency on the scale of seconds and minutes. The detection of these events is a fundamental tool to identify the cyclic alternating patterns (CAPs), the main electrophysiological biomarker of sleep instability.

The CAP is a physiological EEG activity that occurs during NREM sleep and consists of repeated, spontaneous sequences of transient, abrupt frequency/amplitude variations (phase A), which break away from the background activity of the ongoing sleep stage and recur at intervals up to 60 s long. The phase A of a CAP is followed by a return to background activity that is named phase B. Both phase A and B can last between 2 and 60 s, and jointly form a CAP cycle. A succession of cycles defines a CAP sequence, which is usually formed by 5–6 CAP cycles. The absence of CAP for more than 60 s is scored as non-CAP (NCAP), and is considered as a phase of stationary activity (without any phasic CAP-A event), regardless the NREM sleep stage.^2^ Based on the distribution of slow and fast EEG frequencies, A phases are classified in three subtypes: A1, A2, and A3.^3^ These phase A subtypes are not randomly distributed along the night, but instead their appearance is linked with the homeostatic, ultradian, and circadian mechanisms of sleep regulation.^4^^,^^5^

Night sleep is composed of several NREM-REM sleep cycles. Within each cycle is possible to recognize a descending slope, where the sleep stages follow a deepening tendency (from stage N1 to N3), followed by an ascending slope that is characterized by an “ascending” sequence of stages—going from deeper to lighter (from N3 toward stage N1 and then REM sleep).^6^ The CAP contributes to the buildup of the NREM-REM sleep alternation and is considered a precursor of REM sleep, its metrics being essential to understand sleep dynamics. Physiologically, CAP A1 subtypes prevail in the descending branches of the first sleep cycles, and gradually decrease during the night, mirroring the decline of the homeostatic process. Conversely CAP A2 and A3 dominate the ascending branches of sleep cycles, and can be deemed as the forerunners of REM sleep.^5^ Compared to CAP A2 and A3 subtypes, CAP A1 purely consists of slow wave activity (SWA), which refers to oscillations of highly synchronized cortical neurons in the range [0.5−4] Hz. SWA correlates with sleep propensity, which increases in proportion to the duration of wakefulness and decreases in the course of sleep.^7^ Moreover, SWA is involved in producing a sleep-dependent, progressive downscaling of synaptic strength, leading to several benefits in terms of both cellular function and network performance^8^^,^^9^

The sleep architecture described above, with its complex patterns of intermittent activity fluctuations, suggests an underlying tuning to criticality.^10^^,^^11^^,^^12^^,^^13^ Criticality refers to a peculiar state of the system that is characterized by unique properties, such as, long-range correlations, maximal flexibility in response to stimuli, and maximal variability of activity spatiotemporal patterns.^14^^,^^15^^,^^16^ Such properties could be advantageous for the brain to optimize information processing and maximize computational capabilities, and thus achieve optimal functional performance.^17^ Indeed, a number of theoretical and numerical results show that criticality is associated with optimal information processing and computation.^17^^,^^18^^,^^19^^,^^20^ Moreover, reduced flexibility^21^^,^^22^^,^^23^^,^^24^ and breakdown (or alteration) of long-range temporal correlations in brain dynamics^25^^,^^26^ have been associated with neurological diseases and reduced information transfer.

Empirical evidence of brain criticality has been reported across systems, species, and spatial scales.^27^^,^^28^^,^^29^^,^^30^^,^^31^^,^^32^^,^^33^^,^^34^^,^^35^^,^^36^^,^^37^^,^^38^ In particular, empirical observations of scale-free neuronal avalanches—cascades of neural activity exhibiting power-law size and duration distributions—and long-range spatiotemporal correlations in neural activity indicate absence of characteristic temporal and spatial scales in the underlying dynamics, as observed at criticality.^29^^,^^30^^,^^31^^,^^32^^,^^33^^,^^35^^,^^37^^,^^39^^,^^40^^,^^41^^,^^42^^,^^43^^,^^44^ Furthermore, large-scale brain models also predict that the brain is operating close to criticality and permit to reproduce criticality measures (e.g., scaling of the EEG power spectrum) and state transitions,^45^^,^^46^^,^^47^^,^^48^^,^^49^ and the power-law exponents of size and duration distributions of near-critical avalanches can be derived from neural field theory.^47^

Recently, it has been shown that neuronal avalanches during sleep exhibit power law size and duration distributions,^50^^,^^51^^,^^52^ and that sleep may play an important role in tuning the brain to criticality.^53^^,^^54^^,^^55^ At the same time, it has been demonstrated that bursts of dominant cortical rhythms exhibit the hallmarks of self-organized critical dynamics across the sleep-wake cycle of rats, suggesting that criticality could be an essential mechanism for spontaneous sleep-stage and arousals transitions.^11^^,^^12^ However, both the nature of the alleged criticality during sleep and its relationship with alternating patterns of sleep macro- and micro-architecture—in particular the NREM-REM cycle and the CAP—remain poorly understood. On the one hand, the scaling relations among exponents that are expected to hold at criticality have not been verified, and a general framework to understand criticality during sleep is currently missing. On the other hand, the dynamics of avalanches in connection with the highly variable and distinct states composing long- and short-term sleep cycles has not been studied, and the potential functional role of avalanches in sleep regulation has not been explored.

Because of their prominent role as a marker of sleep instability and their key contribution to sleep development (i.e., sleep stage transitions), herein we hypothesize that the CAP cycles, with their alternation of an “active” phase A and a “quiescent phase B”, are a genuine hallmark of tuning to criticality during sleep. Indeed, spontaneous alternation of transient active and quiescent periods is typical of systems that self-organize near a critical point of a non-equilibrium phase transition.^56^^,^^57^ Furthermore, large fluctuations as those involved in the CAP activation phase are a fingerprint of vicinity to criticality.^58^ To test this hypothesis, we study avalanche dynamics in relation to sleep macro- and micro-architecture. First, we characterize the critical behavior of neuronal avalanches during sleep, and determine the scaling relations that connect their critical exponents, showing that they are consistent with a specific universality class. Then, we analyze how avalanche dynamics interacts with the ascending and descending slope of the NREM-REM sleep cycles, and within NREM sleep, how the CAP phases couple with avalanche occurrence. Our analysis shows that avalanche dynamics is closely linked to NREM-REM sleep cycles across night sleep and, crucially, demonstrates that neuronal avalanche occurrence correlates with the activation phase of the CAP (phase A) but is anti-correlated to phase B (quiescent, deactivation phase). These results point to a close relationship between CAP, avalanches, and thus brain tuning to criticality during sleep. We speculate that the CAP may represent the link between preservation of healthy sleep function and self-organization to criticality.

## Results

### Critical exponents and scaling relations for neuronal avalanches during sleep

**Figure 1:**
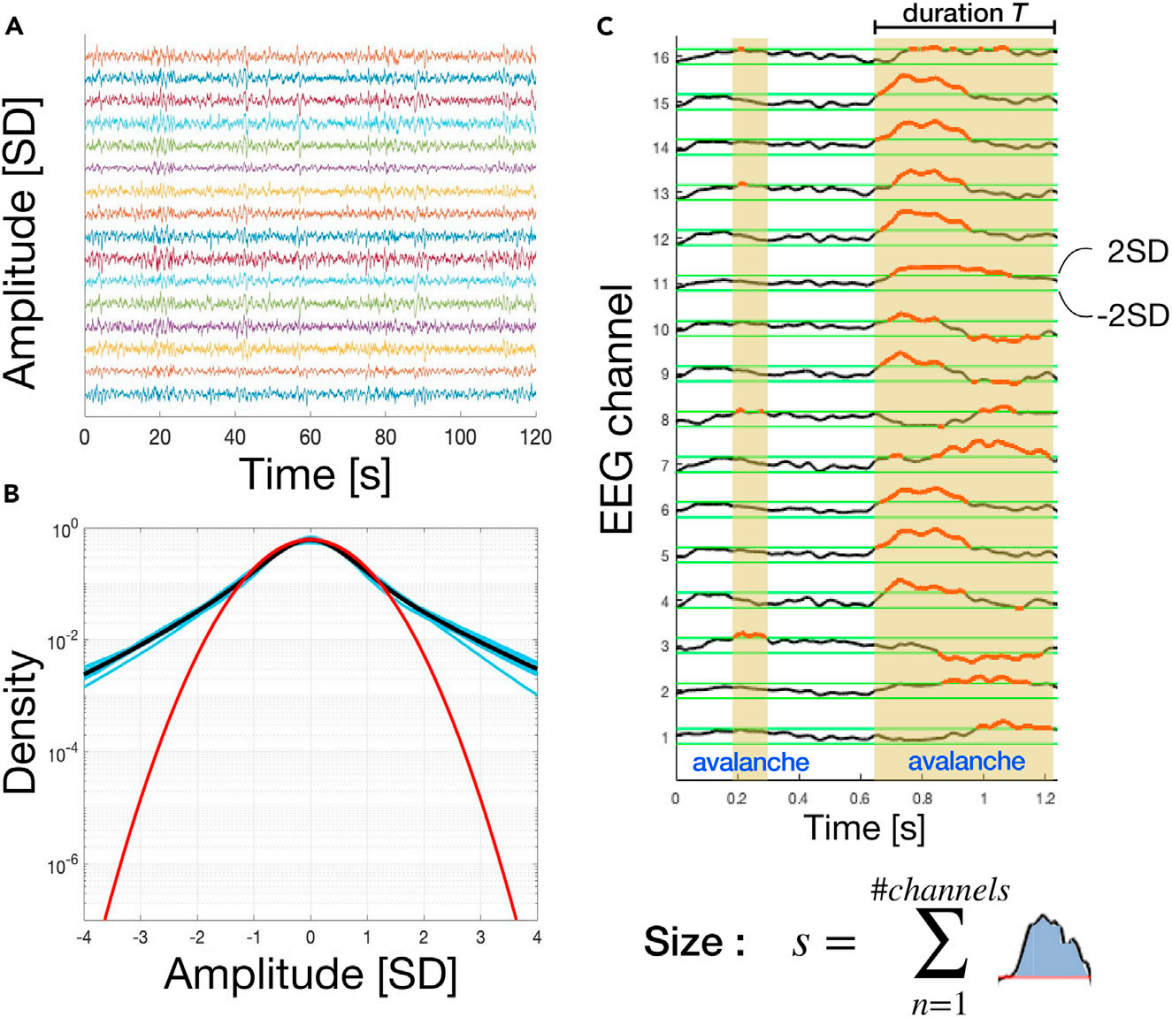
Identification of neuronal avalanches and definition of avalanche size and duration (A) Segments (2 h) of *Z* score normalized EEG signal traces for an individual subject. Each trace corresponds to an EEG channel. (B) Probability density of the *Z* score normalized EEG signal amplitude. The cyan curves in the background are the probability densities for all individual subjects (n = 10 subjects; for each subject we pooled all individual EEG channels). The black curve is the grand average over all subjects. The red curve is the best Gaussian fit for the grand average. We notice that the empirical probability density starts deviating from the Gaussian fit around ±2 SD. (C) A neuronal avalanche is defined as a continuous sequence of signal excursions beyond threshold (red thick line) on one or more EEG channels (upper panel). An avalanche is preceded and followed by periods in which EEG signal are below the threshold in all channels. The size of an avalanche is defined as the sum over all channels of the absolute values of the signals exceeding the threshold (bottom panel).

We analyze brain activity recorded from 10 healthy subjects during night sleep (EEG: 16 channels in three subjects, 19 channels in four subjects and 25 in the remaining three; STAR methods). To measure neuronal avalanches across EEG signals, we first identify prominent deflections in the continuous signal collected from each sensor (Figure 1). We observe that the signal amplitude distributions differ from a Gaussian fit for values larger than 2.0 SD (Figure 1B). Deviations of the EEG amplitude distributions from a Gaussian behavior indicate presence of spatiotemporal correlations that signal the occurrence of clustered, synchronized neural activity.^33^^,^^54^^,^^59^^,^^60^ Thus, for further analysis, we identify large positive and negative signal deflections using an amplitude threshold θ=2 SD (STAR methods; see supplementary information for robustness of avalanche statistics with respect to θ values).

We define an avalanche as a continuous time interval in which there is at least one excursion beyond threshold in at least one EEG channel (Figure 1C, shaded regions). Avalanches are preceded and followed by time intervals with no excursions beyond threshold on any EEG channel.^30^^,^^54^ The size of an avalanche, *s*, is defined as the sum over all channels of the absolute values of the signals exceeding the threshold (Figure 1C, bottom).

**Figure 2:**
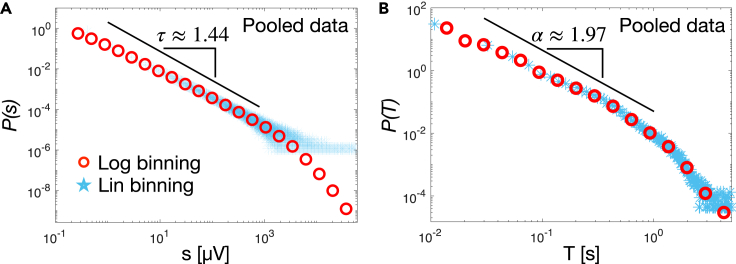
Avalanche size and duration distributions exhibit a robust power law behavior during sleep periods (A) The distribution of avalanche sizes (red circles) follows a power law with exponent τ=1.438±0.001±0.0414 (fit ± error on the fit ± SE; pooled data, 10 subjects). The power law regime is followed by an exponential cut off. The Kolmogorov-Smirnov distance between data and fit is D=0.1, while the log likelihood ratio between the power law and the exponential fit is R = 295 ($p<10^{-5}$). (B) The distribution of avalanche duration follows a power law with exponent α=1.973±0.002±0.0452 (fit ± error on the fit ± SE), followed by an exponential-like cutoff (pooled data, 10 subjects). The Kolmogorov-Smirnov distance between the data and the fit is D=0.07. The log likelihood ratio between the power-law and the exponential fit is R=95 ($p<10^{-5}$). Maximum likelihood estimation of the power law exponents was performed using the Powerlaw Python package^61^ over the range of values indicated by the thick black lines. SE is the systematic error on the fit (STAR methods).

To characterize cortical dynamics underlying sleep macro- and micro-architecture, we identify neuronal avalanches and investigate signatures of criticality across the entire sleep period. To this end, we compute the distribution of avalanche sizes, P(s), and avalanche durations, P(T). In Figure 2 we show the distributions P(s) and P(T) for all subjects (pooled). We find that both the size and duration distributions are well described by a power law, $P\left(s\right)\propto s^{-\tau }$ and $P\left(T\right)\propto T^{-\alpha }$, respectively. In both distributions the power law regime is followed by an exponential cutoff (Figure 2).

Power laws are the hallmark of criticality, and imply absence of characteristic scales in the underlying dynamics.^58^ In this context, the observed power law distributions indicate that neuronal avalanches have no characteristic size and duration, namely they are scale-free. Our analysis shows that the exponent τ for the size distribution is close to 3/2 (τ=1.438±0.001±0.0414; fit ± error on the fit ± SE), while the exponent α for the duration distribution is close to 2 (1.973±0.002±0.0452; fit ± error on the fit ± SE) (Figure 2). The power-law fits were performed over about three decades on the size distributions and two decades on the duration distributions. The limited range of the power-law regime has been associated with finite size effects.^30^^,^^33^ To account for the uncertainty due to the limited fit range, we added a systematic error (SE) to the power law exponent estimates (STAR methods).

**Figure 3:**
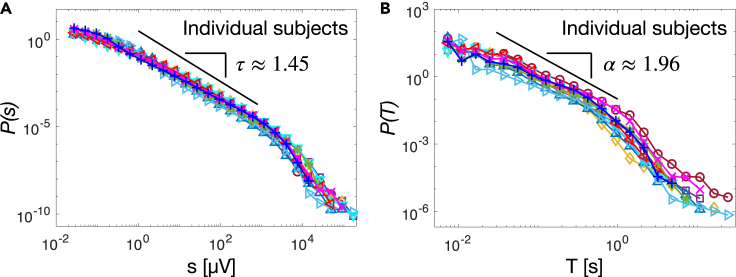
Avalanche size and duration distributions consistently follow a power law behavior across individual subject (A) The distributions of avalanche sizes for individual subjects follow a power law with little variability across subjects (τ=1.45±0.09; mean over subjects ± SD). (B) The distributions of avalanche durations for individual subjects consistently follow a power law, with little variability across subjects (α=1.96±0.16; mean over subjects ± SD). For each individual subject, maximum likelihood estimation of the power law exponents was performed over the range of values corresponding to the thick black line using the Powerlaw Python package^61^.

We compared the power law with an exponential fit by evaluating the log likelihood ratio $R=\ln\frac{L_{p}}{L_{e}}$ between the likelihood $L_{p}$ for the power law and $L_{e}$ for the exponential fit (STAR methods). We found R=295 for the size and R=95 (p value $<10^{-5}$; see STAR methods) for the duration distribution, indicating that the respective power laws better describe the empirical distributions. Importantly, we observe that the power law exponents τ and α are robust and weakly depend on the scale of analysis (supplementary information, Figure S3)—e.g. the threshold θ used to identify avalanches—–, and are consistent across subjects (Figure 3). In Figure 3 we show the avalanche size and duration distributions for each individual subject. Both distributions show little variability across subjects, and follow a power law with exponents τ=1.45±0.03 and α=1.96±0.05 (mean ± SEM). These values are consistent with the values predicted within the mean-field directed percolation (MF-DP) universality class—3/2 and 2, respectively.^62^ We note that the cutoff following the power-law regime appears to deviate from an exponential behavior, particularly for very large avalanche durations (Figure 3B). However, in this range of values, distributions are rather noisy (see error bars in Figure S3), making it difficult to reliably assess the nature of this regime. The analysis of finite size effects shows that the onset of the cutoff depends on the size of the electrodes array (Figure S4), and that the tail shortens significantly for small array sizes, getting progressively closer to an exponential behavior. We cannot exclude that the deviation of the tails from the exponential behavior, observed at large scale with very low probability density ($<10^{-7}$ for the sizes and $<10^{-4}$ for the durations), may be related to intrinsic properties of the data, e.g., dominant, slow delta oscillations.

Moreover, we find that the avalanche branching parameter^22^ is very close to 1, as predicted for critical branching processes (also in the MF-DP universality class), and weakly depends on θ (supplementary information, Figure S3).

**Figure 4:**
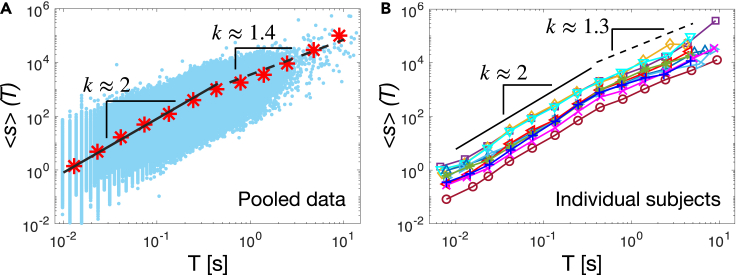
Avalanche sizes and durations are connected by the scaling relationship $\left\langle s\right\rangle \propto T^{k}$ consistent with underlying criticality (A) Average avalanche size as a function of the avalanche duration *T* (red stars; pooled data, 10 subjects). The average avalanche size scales as $\left\langle s\right\rangle \left(T\right)\propto T^{k}$ with k=1.89 for *T*’s within the scaling regime of the distribution P(T). This power-law regime is followed by a crossover to a power-law with a significantly smaller exponent k=1.4 for larger *T*’s. The thick black line is a power law fit for 0.01<T<0.4 s; dashed black line is a power law fit for 0.4<T≤5. Blue dots: (s,T) scatterplot. (B) Average avalanche size as a function of the avalanche duration *T* for all individual subjects. The relationship between avalanche sizes and durations is consistent across subjects, showing a crossover from an exponent k=1.96±0.13, and $k_{1}=1.32\pm 0.19$ (mean ± SD).

Next, we analyze the relationship between avalanche sizes and durations. Near criticality the average avalanche size ⟨s⟩ is expected to scale as a power of the duration *T*, namely $\left\langle s\right\rangle \propto T^{k}$^62^. We find that such a power law relationship holds during sleep (Figure 4). In particular, we observe that, for *T*’s smaller than the duration corresponding to the onset of the cutoff regime in the distribution P(T) (Figures 2 and 3), the average size scales as $\left\langle s\right\rangle \propto T^{k}$ with k≈2 (Figure 4). For larger durations, we observe a crossover to a power law relationship with a smaller exponent k≈1.3 (Figure 4). Importantly, the exponent *k* is robust and independent of the threshold θ used to detect neuronal avalanches (supplementary information, Figure S3). Moreover, we observe that the relation $\left\langle s\right\rangle \propto T^{k}$ is consistent across individual subjects (Figure 4B), the exponent *k* showing little variability across subjects. Specifically, we find k=1.96±0.13 (mean ± SD) for *T*’s smaller than the duration corresponding to the onset of the exponential cutoff in the distribution P(T), and k=1.32±0.19 (mean ± SD) for larger *T*’s (Figure 4B).

Notably, we find that the exponent *k* measured in Figure 4 is in agreement, within errors, with the value predicted by the scaling relation

(Equation 1) $$k=\frac{\alpha -1}{\tau -1}\text{,}$$

which needs to hold at criticality. Indeed, we have that $\frac{\alpha -1}{\tau -1}=2.13\pm 0.26$ (mean ± SEM), and k=1.96±0.04 (mean ± SEM). The scaling relation in Equation 1 has a general validity in avalanche dynamics, as shown in,^63^^,^^64^ where Equation 1 was derived with the only hypothesis that $P\left(s\right)\propto s^{-\tau }$ and $P\left(T\right)\propto T^{-\alpha }$, and that the size fluctuations for fixed durations are small and can be neglected.

In sum, during sleep, the values of the critical exponents τ, α and *k* are very close to the ones predicted for the mean field directed percolation (MF-DP) universality class, with exponents τ=3/2 for the size and α=2 for the duration distribution, and k=2^62^, and the avalanche branching parameter is very close to the critical value σ=1.

### Avalanche dynamics and sleep macro-architecture

We have shown that, during sleep, neuronal avalanches are characterized by a robust scaling behavior in their size and duration distributions (Figures 2 and 3), and that avalanche size and duration are linked by precise scaling relationships (Equation 1; Figure 4). These observations are robust and consistent across subjects, and indicate underlying tuning to criticality during sleep. Next, we investigate the relationship between critical avalanche dynamics, sleep stages, and sleep stage transitions.

We first characterize sleep macro-architecture across all subjects. The main sleep parameters are described in Table 1 (macro-structural measures). The average total sleep time (TST) across the 10 subjects was 423.9 min, with a mean SE of 88.92%. Around 56% of TST was spent in light sleep (N1 = 7.23%, N2 = 48.47%), 23.99% in deep sleep (N3 = 23.99%), and 20.30% in REM. TST for individual subjects is reported in the supplementary information (Table S1).

**Table 1:** Average characteristics of sleep macro-architecture across the analyzed subjects (n = 10)

Measure	MEAN	SD
Sleep latency (minutes)	9,90	12,25
SE (%)	88,92	9,28
TST (min)	423,90	63,31
WASO (min)	40,97	30,46
Stage N1 (min)	28,75	17,47
Stage N1 (%)	7,23	5,20
Stage N2 (min)	207,10	50,78
Stage N2 (%)	48,47	6,67
Stage N3 (min)	99,35	13,40
Stage N3 (%)	23,99	4,90
NREM sleep (min)	335,20	41,67
REM sleep (min)	88,65	32,02
REM sleep (%)	20,30	5,14

For each measure mean and standard deviation (SD) are reported. SE, sleep efficiency; TST, total sleep time; WASO, wake after sleep onset.

**Figure 5:**
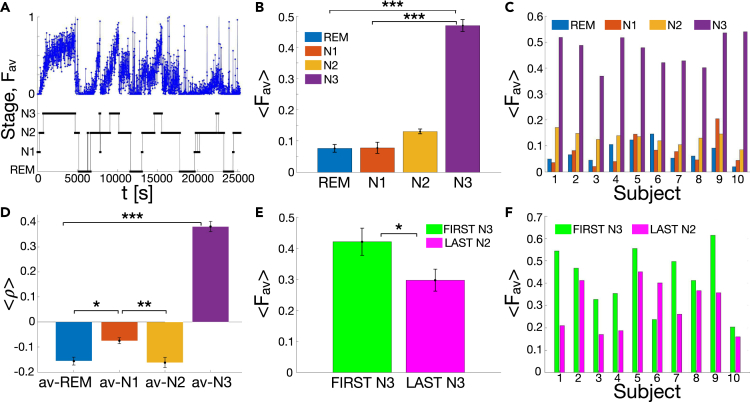
Overnight sleep macro-architecture is associated with strong modulation of avalanche dynamics (A) The density of avalanches (blue dots), $F_{av}\left(t\right)$, is shown as a function of time, together with the corresponding sleep stages and sleep stage transitions (REM, N1, N2, N3 black line) for an individual subject. $F_{av}\left(t\right)$ increases gradually in N2 and N3, and then abruptly decreases when transitioning from N3 to either N2, N1 or REM. Waking periods during sleep have been removed. (B) Mean avalanche density for each sleep stage (REM, N1, N2, N3) averaged across subjects. The density $F_{av}\left(t\right)$ is highest in N3 and gradually decreases for N2, N1, and REM (one-way ANOVA on ranks: $p=5\cdot 10^{-6}$; significant pairwise comparison: N3 vs. REM ($p=7\cdot 10^{-5}$), N3 vs. N1 ($p=3\cdot 10^{-5}$). (C) Mean avalanche density for each sleep stage and for each individual subject. The behavior observed for the group average is consistent across individual subject, N3 being the sleep stage with the highest density of avalanches. (D) The mean Pearson correlation coefficients $\rho_{x,y}$ (see Equation 2) between avalanche occurrence and sleep macro-architecture (namely REM, N1, N2, N3) shows that avalanches tend to occur mostly during N3 (One-way ANOVA group comparison: $p=3\cdot 10^{-23}$). Pairwise differences between N3 and all other sleep stages are significant (N3 versus N2: $p=10^{-21}$; N3 versus N1: $p=3\cdot 10^{-19}$; N3 versus REM: $p=10^{-21}$). N2 is significantly different from N1 (p=0.006), and N1 is significantly different from REM (p=0.013). (E) Mean density of avalanches in the first and last N3 stage of the recordings averaged over all subjects. We observe that the density is significantly higher during the first N3 (p=0.04). (F) Avalanche density in the first N3 (blue) and last N3 (red) for each individual subject. The density is higher in the first N3 for all subjects but the subject #6. Error bars indicate the SE. Significance legend: ∗∗∗ for p<0.001; ∗∗ for p<0.01; ∗ for p<0.05. The ∗∗∗ in panel D refers to the pairwise comparison between N3 and all the other sleep stages. Differences are not significant where no stars are reported.

To study the interplay between sleep macro-architecture and avalanche dynamics, we introduce the avalanche density, $F_{av}\left(t\right)$, defined as the amount of time occupied by avalanches in a sliding window of length $u_{0}$ (STAR methods), and study the temporal evolution of $F_{av}\left(t\right)$ in relation to the sleep macro-architecture. In the following we fix $u_{0}=10$ s, which approximately corresponds to the largest avalanche duration we observed (Figures 2 and 3). In Figure 5A we show the avalanche density $F_{av}\left(t\right)$ as a function of time for an individual subject, together with the corresponding hypnogram. We observe that $F_{av}\left(t\right)$ gradually increases in parallel with sleep deepening, i.e., going from REM to N1, N2, and finally N3: $F_{av}$ is very small during stage N1, reaches an intermediate value during stage N2, and increases substantially during stage N3, where it peaks slightly before the following transition back to N2 and REM (Figure 5A). Although the avalanche density tends to decrease across the night and is, on average, much smaller at the end of the night, we find that this trend repeats throughout the night in correspondence to the descending REM → N3 of the NREM-REM sleep cycle. In contrast to this gradually increasing trend, we observe that the avalanche density decreases rather abruptly with transitions from N3 to N2 and N1—the ascending phase of the NREM-REM sleep cycle. In sum, we find that the avalanche density gradually increases during the descending slope of each sleep cycle, whilst it rapidly decreases in the ascending slope of the same cycles that precedes the onset of REM sleep (Figure 5A).

Our analysis shows that the density of avalanches is significantly higher during N3 as compared to N1 and REM (Figures 5B and 5C), while the pairwise comparison between N3 and N2 shows non-significant differences (p=0.06; STAR methods). The analysis of the Pearson correlation coefficient $\rho_{x,y}$ (STAR methods, Equation 2) shows that avalanche occurrence, on average, is positively correlated with N3, the deepest sleep stage, while it is either weakly or slightly negatively correlated with other sleep stages (Figure 5D). Finally, we observe that, during N3, the avalanche density tends to increase with time (Figure 5A).

Interestingly, we observe that, for relatively low densities ($F_{av}<0.4$.), both the number of avalanches, $N_{av}$, and the mean duration of avalanches, ⟨T⟩, in the sliding window $u_{0}$, tend to increase with the density $F_{av}$ (supplementary information, Figure S6). On the other hand, larger densities do not correspond to a consistent increase in $N_{av}$, but are rather associated to longer avalanches. Because $F_{av}>0.4$ mostly occur during N3 sleep stage (see Figures 5A and 5B), this indicates the presence of longer avalanches during N3, which could be related to dominance of slow delta oscillations in this stage (see definition of avalanches; Figure 1 and STAR methods). On the other hand, the gradual increase of density from the low values in N1 to the intermediate values in N2 is accompanied by a gradual increase of the number $N_{av}$ of avalanches.

Importantly, we notice that the avalanche density peak—typically located within N3 periods—is higher in the first half of the night, d progressively decreases during the second half of the night. To quantify the significance of this behavior with respect to the characteristics of neuronal avalanches, we compare the avalanche density, as well as avalanche size and duration distributions, in the first and last N3 stage of the sleep recordings. We find that avalanche size and duration distributions in the first N3 are comparable to the distributions calculated in the last N3 (SI, Figure S5). Furthermore, the scaling relation $\left\langle s\right\rangle \propto T^{k}$ between avalanche size and duration is satisfied both in the first and last N3, with the same values of the exponent *k* (SI, Figure S5). On the other hand, we observe that the avalanche density is significantly higher during the first N3 as compared to the last N3 (Figures 5E and 5F) (*t*-test: p=0.04). This is consistent across subjects (Figure 5F), with the exception of subject #6 for which we observe that the density is higher in the last N3. Such deviation from the average behavior may be related to general differences we observed in sleep of subject #6. For instance, this subject presented an unusually short duration of the N3 stage at the beginning of the night, followed by a gradual increase of N3 in the second half of the sleep.

### Avalanche dynamics and sleep micro-architecture

The analysis of the avalanche density across sleep stages has shown that neuronal avalanches tend to occur with higher frequency during NREM sleep. However, NREM sleep has a complex micro-architecture that is characterized by the CAP (see supplementary information, Figures S1 and S2 for a representative example of CAP oscillations and NCAP during NREM sleep).^3^ In our data, the mean CAP rate was 49.19% with the following distribution across NREM stages: N1 = 41.69%, N2 = 48.36%, and N3 = 53.37% (Table 2). On average, subjects presented 37.1 CAP sequences per night, with a mean duration of 4.55 min. With respect to CAP subtypes distribution, 206 were A1 (25.7% of the CAP time); 67.2 were A2 (9.2% of the CAP time), and 83.8 were A3 (14.19% of the CAP time). A1’s were more present during stage N3 (50.21%) as compared to N2 (5.72%) and N1 (1.49%), in agreement with previous studies.^65^ On the other hand, subtypes A2 and A3 predominated in stage N1 (particularly A3, 37.77%) and N2 (14.39% for A2 and 17.46% for A3) (Table 2).

**Table 2:** Average characteristics of sleep micro-architecture across the analyzed subjects (n = 10)

Measure	Mean	SD
CAP time (minutes)	162,51	42,3
CAP rate (%)	49,05%	0,1
CAP sequences (n)	37,1	8,3
CAP sequence length (min)	4,55	1,6
CAP cycle (n)	357,6	104,1
Phase A length (s)	8,59	1,4
Phase B length (s)	20,67	3,5
Subtype A1 duration (s)	6,42	2,0
Mean time subtype A1 (%)	25,7	11,1
Subtype A2 duration (s)	8,63	2,0
Mean time subtype A2 (%)	9,29	5,8
Subtype A3 duration (s)	12,72	1,32
Mean time subtype A3 (%)	5,76	1,9

**Figure 6:**
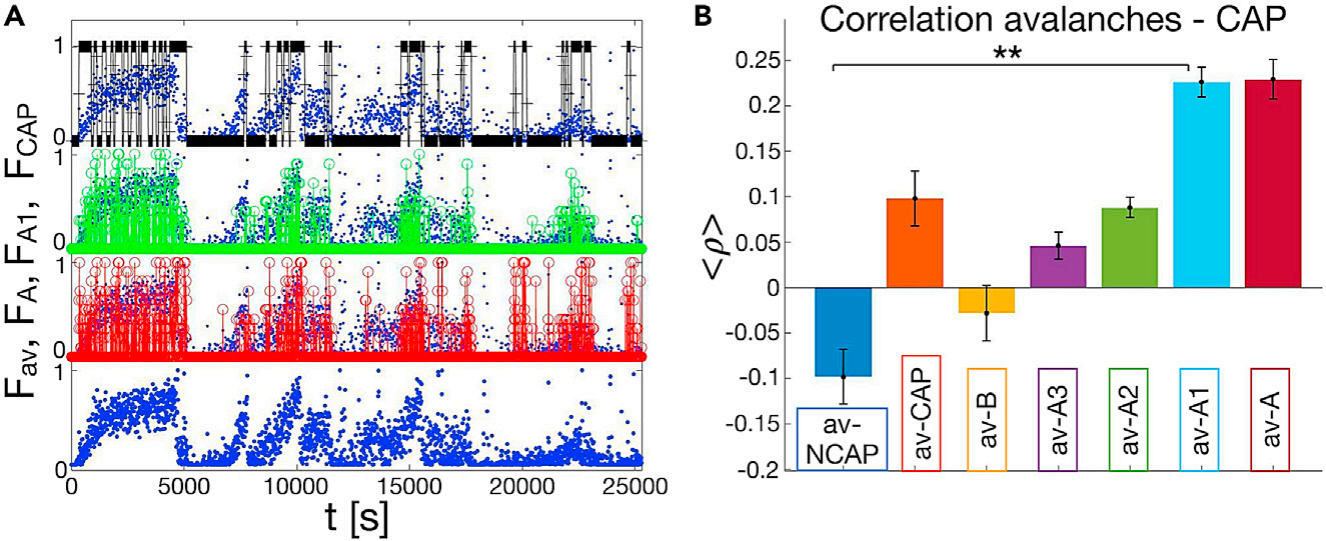
Occurrence of neuronal avalanches is coupled with the occurrence of the CAP (A) Density of avalanches versus density of CAP phases as function of time for an individual subject. Density of avalanches in blue, density of phase A in red, density of phase A1 in green, density of CAP in black. (B) The mean Pearson correlation coefficients ρ(x,y) (average over subjects; see STAR methods, Equation 2) between avalanche occurrence and micro-architecture features (NCAP, CAP, B, A, and A subtypes A3, A2, A1). Error bars indicate the SE. A one-way ANOVA test shows that group differences are significant ($p=9\cdot 10^{-16}$). Pairwise comparisons show significant differences for all couples (p<0.02) but av-NCAP vs. av-B, av-CAP vs. av-A3, av-CAP vs. av-A2, av-B vs. av-A3, av-A2 vs. av-A3, and av-A1 vs. av-A. Significance legend: ∗∗∗ for p<0.001; ∗∗ for p<0.01; ∗ for p<0.05. The ∗∗ in panel B refers to the pairwise comparison between av-A1 and all the other bars but av-A. Differences are not significant where no stars are reported.

To dissect the relationship between CAP and occurrence of neuronal avalanches during NREM sleep, we compare the time course of the avalanche density with the density of distinct CAP phases (Figure 6A) defined as $F_{X}\left(t\right)=\left(u_{X}\left(t\right)\right)/u_{0}$, where X denotes the specific CAP phase—A, A1, A2, A3, B—and $u_{X}\left(t\right)$ the time occupied by the specific CAP phase in a window of length $u_{0}=10$ s. We observe a remarkable time correspondence between the temporal profile of the density of avalanches $F_{av}\left(t\right)$ and the density of CAP, with the peaks in avalanche density corresponding to high density of CAP—in particular phase A and A1 (Figure 6A). Specifically, we notice that, with sleep deepening, the progressive increase of CAP density is accompanied by a parallel increase in avalanche density. We find that the percentage of phase A occupied by neuronal avalanches is about 42.16%, while the percentage of sleep time occupied by avalanches is 19.21% (STAR methods). Interestingly, CAP phase A1 is even richer in avalanches compared to CAP A phases A2 and A3 (53.32% versus 43.84% and 27.72%, respectively).

The physiological increase of CAP cycles during N2 and N3, indirectly leads to a reduction of time occupied by NCAP sleep. Furthermore, during the deepest stages of NREM sleep, CAP’s typically present shorter phases B. These changes in the sleep micro-dynamics lastly sustain the observed increase of avalanche density.

Next, we measure the Pearson correlation coefficients between occurrence of neuronal avalanches and different CAP phases (see STAR methods, Equation 2). We find positive correlations between occurrence of avalanches and CAP phase A, in particular CAP phase A1 (Figure 6B). On the contrary, we observe negative correlations between occurrence of avalanches, CAP phase B, NCAP periods. This indicates that the occurrence of avalanches during NREM sleep is strictly related to occurrence of CAP, and in particular CAP phase A1. These results are consistent across subjects, as shown in Table 3.

**Table 3:** Pearson correlation coefficient between avalanche occurrence and CAP subtypes for the analyzed individual subjects (n = 10) (STAR methods)

Subject	av-NCAP	av-CAP	av-B	av-A3	av-A2	av-A1	av-A
#1	−0.240	0.240	0.113	0.050	0.080	0.270	0.260
#2	−0.140	0.140	−0.016	0.130	0.150	0.220	0.290
#3	−0.060	0.060	−0.041	0.090	0.090	0.170	0.190
#4	−0.140	0.140	0.039	0.010	0.110	0.230	0.190
#5	−0.040	0.040	−0.013	0.000	0.050	0.190	0.130
#6	−0.020	0.020	−0.206	0.040	0.090	0.250	0.270
#7	−0.070	0.070	−0.074	0.080	0.110	0.200	0.240
#8	−0.220	0.220	0.056	0.040	0.110	0.330	0.340
#9	−0.140	0.140	0.016	0.050	0.060	0.240	0.230
#10	0.080	−0.080	−0.158	−0.030	0.030	0.160	0.130
Mean	−0.099	0.099	−0.029	0.046	0.088	0.226	0.227
Std error	0.030	0.030	0.030	0.015	0.011	0.016	0.021

## Discussion

In this paper we analyzed the scaling properties of neuronal avalanches during sleep in healthy volunteers, and investigated the relationship between avalanche dynamics and sleep macro- and micro-architecture, with a particular focus on the CAPs. We showed that the scaling exponents characterizing neuronal avalanches are consistent with the MF-DP universality class, and obey the theoretically predicted scaling relations. This indicates that, during physiological sleep, brain dynamics is consistent with criticality and is satisfactorily described by the MF-DP universality class. Furthermore, we introduced a measure—the density of avalanches—to quantify the relationship between avalanche dynamics and sleep macro- and micro-architecture. Our analysis showed that the distribution of avalanches in time is not random but closely follows the descending and ascending phase of the NREM-REM cycles. Within such cycles, the presence of neuronal avalanches is linked to the occurrence of CAP during NREM sleep. Specifically, we found that the density of avalanches is higher during NREM, and, within NREM sleep, avalanche occurrence is positively correlated with the phase A of the CAP, in particular the phase A1. This suggests a close relationship between modulation and control of brain criticality, sleep macro- and micro-architecture, and brain function, which we discuss in turn.

### Brain dynamics and criticality during sleep

Empirical evidence indicates that the human brain operates close to a critical regime both in resting wakefulness and during sleep.^11^^,^^12^^,^^35^^,^^50^^,^^51^^,^^52^ In particular, recent studies suggest that criticality plays a key role in determining the temporal organization of sleep stage and arousal transitions,^11^^,^^12^ and sleep deprivation progressively disrupts signatures of criticality^54^ and alters brain connectivity.^66^ However, critical dynamics during sleep remains poorly understood. In this respect, a key open question concerns the universality class to which brain criticality obeys during sleep. The importance of knowing the universality class resides in the possibility of predicting properties and behaviors that are not easily accessible otherwise, or even expected, opening the way to further experimental and theoretical development. This is made possible by a set of scaling relationships connecting the critical exponents. Specifically, for the directed percolation (DP) universality class there are only three independent exponents, and all others can be derived from known scaling relationships.^67^ For systems out of equilibrium, as the brain, the DP universality class is expected to describe any absorbing phase transition—i.e., a transition between an active and an inactive (absorbing) state—with just one absorbing state (or more but non-equivalent). Systems with more than one equivalent absorbing state falls in different universality classes, and thus are described by a different set of exponents.^68^

To the best of our knowledge, this is the first study exploring the scaling relations among critical exponents of neuronal avalanches during sleep. We reported a picture that is consistent with the MF-DP universality class. Indeed, we have shown that (1) the critical exponents for the avalanche size and duration distributions are very close to the prediction of MF-DP universality class, i.e., τ=3/2, α=2, respectively; (2) the exponent *k* connecting sizes and durations is very close to 2, as predicted; (3) the exponents τ, α, and *k* correctly satisfy the expected scaling relation. The fact that sleep criticality in healthy subjects seems to be in line with the MF-DP class is important to assess deviations from criticality in pathological sleep, and a key step toward sleep biomarkers based on EEG criticality measures.

The exponent *k* has been previously measured in the awake resting-state, from zebrafish and rats to monkeys and humans.^37^^,^^42^^,^^44^^,^^69^^,^^70^^,^^71^^,^^72^ In line with our findings, recent works in awake nonhuman primates^69^ and awake mice^44^ found that k≃2 in the range corresponding to the power law regime of the size and duration distributions, and k∈[1,1.5] only in the region that corresponds to the cut-off of the distributions—where we found k≃1.3. Similar results were found in zebrafish.^37^ Deviation from the MF-DP value k=2 was observed in the resting-state of the human brain,^71^ in *ex vivo* turtle visual cortex,^73^ in the barrel cortex of anesthetized rats,^42^ in cortex slice cultures,^74^ and in freely behaving and anesthetized rats.^70^ Sub-sampling in brain activity recordings has been suggested as a potential origin of such deviation from the theoretically predicted value,^75^^,^^76^ and could affect scaling exponents in our analysis (we have between 16 and 25 electrodes). Alternatively, a recent work has shown that, in a 2D Wilson-Cowan neural network, the value of the exponent *k* is related to the network connectivity, with k≃1.3 for a 2D connectivity, and k=2 when the mean-field approximation holds.^77^ This may suggest that, in our case, the mean-field approximation is justified for relatively small avalanches involving few electrodes, but not for large-scale EEG avalanches. However, to verify whether this is due to the structure of the underlying, collective neural activity, simultaneous multi-scale recordings would be needed. In this respect, an important open question is the relationship between avalanches across scales (from spike avalanches to EEG/MEG)—a key question to correctly interpret discrepancies in scaling exponents across experiments. Alternatively, a recent work has shown that, in a 2D Wilson-Cowan neural network, the value of the exponent *k* is related to the network connectivity, with k≃1.3 for a 2D connectivity, and k=2 when the mean-field approximation holds.^77^ This may suggest that, in our case, the mean-field approximation is justified for relatively small avalanches involving few electrodes, but not for large-scale EEG avalanches. However, to verify whether this is due to the structure of the underlying, collective neural activity, simultaneous multi-scale recordings would be needed. In this respect, an important open question is the relationship between avalanches across scales (from spike avalanches to EEG/MEG)—a key question to correctly interpret discrepancies in scaling exponents across experiments. Moreover, the presence of a large-avalanche regime with k≃1 after a regime with k≃2 has been also observed at the critical point, near a bistability regime in a stochastic Wilson-Cowan model whose function of activation mimics cooperative effects.^78^ In this model, the regime with k≃1 is related to closeness of the system operating point to an underlying self-sustained regime. In sum, together with subsampling and coarse-graining effects, further investigation is needed to understand the crossover from k≃2 to k≃1.

### Neuronal avalanches and sleep macro-architecture

Analyses of scalp EEG and human intracranial depth recordings have shown that avalanche size and duration distributions follow a similar power law behavior across the sleep-wake cycle, with exponents in line with our observations.^50^^,^^52^ Similarly, the analysis of whole-brain fMRI data have confirmed a critical (or near-critical) behavior from wakefulness to deep sleep, with little differences in the power-law exponent of the avalanche size distribution (in particular between wakefulness and stage N2).^51^

On the other hand, here we have shown that, although the static properties remain fairly stable across different sleep stages,^50^^,^^51^^,^^52^ avalanche dynamics is modulated by the ascending and descending slope of the NREM-REM sleep cycles. By analyzing the temporal evolution of the avalanche density, we found that avalanche occurrence markedly and progressively increases with NREM sleep stages N2 and N3 and, specifically, during periods of sleep deepening (descending slope of sleep cycles), in parallel with the increase of SWA. On the contrary, the abrupt decrease in avalanche density during the ascending slope of sleep cycles suggests a negative influence from REM-on/wakefulness circuits with respect to their appearance. The different behavior of avalanche density during the descending and ascending slopes of the sleep cycles was not previously observed, despite the crucial role of such dynamics for sleep regulation. In terms of sleep physiology, the descending and ascending slopes of sleep cycles are markedly different: during the descending slope, sleep-promoting forces, i.e., homeostatic sleep propensity, are stronger,^79^^,^^80^ the thalamocortical system works in the burst-firing mode and brainstem cholinergic pathways are tonically repressed. Conversely, during the ascending slope, the NREM driving forces become weaker, sleep is more vulnerable toward pro-arousal intrusions and REM-promoting outputs prevail.^65^ Taking this into account, our results suggest that avalanche occurrence is not random across the sleep cycles, but instead contributes to define the dynamical interplay between sleep-wake promoting networks.

The reported scale-free avalanche activity within the NREM sleep coexists with SWA which, in contrast, has a characteristic timescale. The coexistence of scale-free and scale-specific brain activity, and specifically the relationship between neuronal avalanches and oscillations, is still poorly understood and deserves further investigation. In a recent human study, it has been shown that the dynamics of avalanches depends on the dominant, coexisting brain oscillations, and differs between awake resting-state and NREM sleep.^60^ During resting wake, alpha oscillations induce attenuation of avalanches (i.e., consecutive sizes tend to be smaller) within a single alpha cycle, and corresponding amplification over several alpha cycles (i.e., consecutive sizes tend to be smaller). In contrast, during NREM sleep, only the attenuation regime has been observed, both at short and long timescales.^60^

The coexistence of avalanches and oscillations has been also investigated in rodents and in mature cortex slice cultures.^81^^,^^82^^,^^83^ In rodents, nested θ and β/γ oscillations were found to be embedded in neuronal avalanches,^81^ while in cortex slice cultures a hierarchical organization of avalanches in relation to θ and γ oscillations was described.^82^

From a theoretical perspective, such a coexistence has been studied in a few models,^71^^,^^84^^,^^85^^,^^86^^,^^87^ and quantitatively captured in the resting human brain by an adaptive Ising model indicating that the coexistence of oscillations and avalanches in brain activity occurs close to a non-equilibrium critical point at the onset of self-sustained oscillations.^71^^,^^88^ Yet, the precise relationship between collective oscillations and avalanches remains an open question.

### Avalanches and sleep micro-architecture

Sleep architecture is composed of numerous oscillatory patterns, including, above all, the CAP.^89^ CAP’s occur on time scales of seconds or minutes, accompany sleep stage shifts, and contribute to the organization of sleep cycles. Our analyses demonstrated positive correlations between CAP and avalanche occurrence, and negative correlations for NCAP sleep. Such link suggests a close relationship between CAP and brain tuning to criticality during sleep, a key aspect that should be further investigated in future work.

Although the definition of avalanches (large, collective non-gaussian fluctuations of brain activity) is not related to the definition of CAP phase A, our results show that neuronal avalanches are correlated with the occurrence of CAP phase A. In particular, we observed stronger correlations between avalanche occurrence and the CAP A1 subtype, and weaker positive correlation with subtypes A2 and A3. Interestingly, the correlation between avalanches and the phase A of the CAP is more prominent than the correlation with the CAP itself— phase A and phase B together. We speculate that this could be due to the opposite significance of CAP phase A and B with respect to sleep dynamics. Electrophysiologically the phase B is characterized by the rebound of background EEG activity after the strong “activation” driven by the phase A. Compared to phase A, the phase B could be described as a “lower arousal reaction” or mechanism of deactivation.^90^ Phases B reflect a period of transient inhibition and have been associated with nocturnal sleep apnea in patient with sleep-breathing disorder as well as with inhibitory effect on nocturnal epileptiform discharges in patients with generalized epilepsies.^89^^,^^91^ Importantly, we did not observe significant correlation between avalanche occurrence and phase B, corroborating our assumption about the relationship between CAP phase A and avalanches. The prominent correlation between avalanche occurrence and CAP “activation phase” A1 may suggest that neuronal avalanches emerge at the edge of a synchronization phase transition, as recent numerical studies indicate.^85^^,^^86^^,^^92^

Finally, we note that CAP-A1 physiologically prevail in the first half of the night and during the descending slope of each sleep cycle, boosting or maintaining SWS. Similarly, the avalanche density decreases moving from the first to the last sleep cycle. Hence, both CAP phase A and neuronal avalanches follow a physiological, homeostatic decay throughout the night.

### Neuronal avalanches, CAP, and learning mechanisms: An intriguing hypothesis

Sleep is crucial to renormalize synaptic weight, ensure an optimal and effective network state for information processing, and preserve cognition.^9^ Renormalization of synaptic weights taking place during sleep may serve to keep the network close to criticality.^93^ In line with this view, the here reported higher concentration of avalanches during SWS and CAP-A1 indicate that these states may exert a pivotal role in modulating and restoring brain criticality. Furthermore, because CAP-A1 has been proposed to play a role in the sleep-dependent learning processes,^8^ our observations point to a functional link between critical avalanche dynamics and sleep-dependent learning processes, as shown in recent numerical studies.^92^^,^^94^ Specifically, it has been demonstrated that, within the alternation of up- and down-states observed during SWS, the sequence of avalanches occurring in the up-states correspond to an intermittent reactivation of stored spatiotemporal patterns, a mechanism that is key for memory consolidation.^95^

### Conclusions

Overall, our findings open a novel perspective on the relationship between critical brain dynamics and physiological sleep. We provided a comprehensive account of the critical exponents and scaling relations for neuronal avalanches, demonstrating that brain dynamics during sleep is consistent with the MF-DP universality class. This sets the bases for future investigation of neural collective behaviors occurring during sleep, including their functional role in relation to criticality. As a first step in this direction, our study provides evidence of a functional link between avalanche occurrence, slow-wave sleep dynamics, sleep stage transitions and occurrence of CAP phase A during NREM sleep. As CAP is considered one of the major guardians of NREM sleep^96^ that allows the brain to react dynamically to any external perturbation and contributes to the cognitive consolidation processes occurring in sleep, we speculate that neuronal avalanches at criticality might be associated with flexible response to external inputs and to cognitive processes—a key assumption of the critical brain hypothesis. This is a crucial aspect that should be investigated in future work—particularly with respect to the hypothesized relationship between tuning to criticality and sleep architecture.^10^^,^^11^^,^^12^ Moreover, based on our results, one could speculate that a relationship between occurrence of neuronal avalanches and physiological sleep measures exists. To address this point, additional studies in pathological sleep conditions where both CAP and criticality-based metrics show a deviation from the physiological parameters are needed.^96^^,^^97^

### Limitations of the study

The question of the universality class of neuronal avalanches, both in sleep and wake, is still debated.^44^^,^^70^^,^^76^^,^^86^ Although our results show that neuronal avalanches during sleep are described by scaling exponents and scaling relationships consistent with the MF-DP universality class, this is not sufficient to prove that during sleep neuronal avalanches belong to the MF-DP universality class. The main reasons are: (1) although most non-equilibrium processes with an absorbing phase transition belong to the DP universality class, we cannot exclude, based on our estimates of the exponents, that neuronal avalanches do not belong to a different universality class with similar mean-field exponents, e.g., the mean-field dynamical percolation.^56^^,^^67^ Further investigation of this point would benefit from multi-scale recordings to measure other critical exponents that could confirm (or reject) the MF-DP as the universality class for neuronal avalanches; (2) we define neuronal avalanches as spatiotemporal sequences of threshold-crossing events across EEG sensor array—a sort of coarse-grained measure—but we do not have access to the actual activity of underlying neural populations and, thus, we cannot prove that the same results hold for spike avalanches. Simultaneous multiscale recordings would be needed to demonstrate consistency with the MF-DP across scales, also taking into account potential subsampling effects.^75^ Indeed, in the awake state, deviation from the MF-DP exponents in spike avalanches in mice^70^ and in zebrafish^37^ has been reported. However, this deviation may arise from subsampling issues.^98^

Finally, the relatively small number of subjects requires a word of caution about the presented results. Although our individual recordings are rather long (424±63 min, Table 1) and provide a fairly robust individual statistics for avalanches ($>10^{5}$ avalanches per subject), sleep stages (NREM: 335±42 min; mean ± SD), and CAP (CAP time: 162±42 min; mean ± SD), our cohort comprises 10 subjects with a relatively wide age range—from 28 to 53 years. This limits the statistical power of our analysis and, in particular, does not allow stratification of results according to age, which may be relevant in this context. We observed that results for avalanche dynamics were robust across subjects (Figures 3 and 4; Figure S7), with small variability in the scaling exponents. Furthermore, we found that avalanche size and duration distributions of the youngest and oldest subjects (28 vs. 53 years) are very close to each other, showing no larger differences than those observed between the two youngest subjects (28 years) (Figure S7). Similarly, we observed that the relationship between avalanches and sleep architectures is consistent across subjects (Figures 5B and 5C; Table 3), with no apparent age-related effects when comparing the youngest and oldest subjects (Figure S8). However, because of the limited number of subjects, we cannot exclude that avalanche dynamics and its relationship with sleep architecture depends on aging. Future work on larger cohorts—including a significant number of healthy subjects in each age group—is needed to address this key point, as well as to avoid potential small-sample biases (e.g., due to the intrinsic nature of the EEG) and thus confirm the presented results.

## STAR★Methods

### Key resources table

**Table:** 

REAGENT or RESOURCE	SOURCE	IDENTIFIER
**Software and algorithms**		

MATLAB R2020b	Mathworks	www.mathworks.com
Python version 3.1	Python Software Foundation	www.python.org
powerlaw	Alstott et al.^61^., PLoS ONE 9 (1): e85777	https://github.com/jeff alstott/powerlaw

**Other**		

EEG of the human brain	this paper	

### Resource availability

#### Lead contact

Further information and requests for resources and reagents should be directed to and will be fulfilled by the lead contact, Silvia Scarpetta (sscarpetta@unisa.it).

#### Materials availability

This study did not generate new unique reagents.

### Experimental model and study participant details

The data analyzed in this study were extracted from overnight polysomnographic (PSG) recordings acquired from the Parma (Italy) Sleep Disorders Center database. Ten healthy subjects, 5 males and 5 females, mean aged 39,6 years (age range 28–53), were selected after the accomplishment of an entrance investigation. Subjects were selected based on the following inclusion criteria: (i) absence of any psychiatric, medical and neurological disorder (ii) normal sleep/wake habits without any difficulties in falling or remaining asleep at night: a personal interview integrated by a structured questionnaire confirmed good daytime vigilance level; (iii) no drug intake at the time of PSG and the month before; (iv) full night unattended PSG recordings performed with EOG (2 channels), EEG [Ag/AgCl electrodes placed according to the 10–20 International System referred to linked-ear lobes]. Recording electrodes were 16 in three subjects (subject #1, #7, #9; Fp2, F4, C4, P4, O2, T4, T6, Cz, Pz, Fp1, F3, F7, P3, O1, T3, T5); 19 in four subjects (subjects #5, #6, #8, #10; Fp2, F4, C4, P4, O2, F8, T4, T6, Fz, Cz, Pz, Fp1, F3, C3, P3, O1, F7, T3, T5); 25 in three subjects (subject #2, #3, #4; CP3, CP4, C5, C6, C2, 573 C1, FC4, FC3, F4, C4, P4, O2, F8, T4, T6, Fz, Cz, Pz, F3, C3, P3, O1, F7, T3, T5). EMG of the submentalis muscle, ECG, and signal for SpO2 (pulse-oximetry O2-saturation) were recorded.

### Method details

#### Data acquisition and pre-processing

PSG recordings were acquired using a Brain Quick Micromed System 98 (Micromed, SPA). A calibration of 50 μV was used for EEG channels with a time constant of 0.1 s and a low-pass filter with 30 Hz cut-off frequency. EEG sampling rate was 256 Hz for six subjects while for the remaining four cases, one was recorded using a sampling rate of 128 Hz (subject #1) and the remaining three (subject #2, #3, #4) using 512 Hz. Each signal was recorded and examined by an expert clinician (CM, IT, LP). Analysis of sleep recordings (see Section method details) was performed with Embla RemLogic Software. The institutional Ethical Committee Area Vasta Emilia Nord approved the study (protocol nr. 19750).

#### Analysis of sleep macro-architecture

Sleep was scored visually in 30-s epochs using standard rules according to the American Academy of Sleep Medicine (AASM) criteria.^99^ Conventional PSG parameters included total time in bed (TIB) (minutes), total sleep time (TST) (minutes), sleep latency (minutes), rapid eye movement (REM) latency (minutes), sleep efficiency (%), wake after sleep onset (WASO) (minutes), as well as percentage of NREM (N1, N2, N3) and REM stages. Acquisition lengths correspond to the TST which is defined by total amount of sleep time scored during the total recording time. The Table S1 SI summarizes the TST for each subject.

#### Analysis of sleep micro-architecture

Sleep micro-architecture evaluation refers to the quantification of CAP parameters based on the published international atlas,^3^ and was manually performed using Embla REM-logic software by somnologists with strong expertise in the field (LP, CM). CAP is a global EEG phenomenon involving extensive cortical areas, thus CAP phases should be visible on all or most EEG leads. CAP is characterized by the alternation of phase A (transient electrocortical events) and phase B (low voltage background), both lasting between 2 and 60 s. According to published criteria^3^ phase A activities were classified into three subtypes.

1. Subtype A1. EEG synchrony in the lower frequency (theta-delta) is the predominant activity and the EEG desynchrony (mixed frequency ranges, mainly alpha and beta) occupies <20% of the whole phase A. Subtype A1 may include delta burst, K-complex sequences, vertex sharp transients, polyphasic bursts with <20% of EEG desynchrony.
2. Subtype A2. It is a mixture of fast and slow rhythms where the EEG desynchrony in high frequency range occupies 20−50% of the entire phase A. This subtype includes polyphasic bursts with 20−50% of high frequency activity.
3. Subtype A3. EEG desynchrony alpha-beta is the predominant activity (>50%) of the phase A. Subtype A3 includes K-alpha, EEG arousal and polyphasic bursts with >50% of high frequency activity.

The percentage of NREM sleep occupied by CAP sequences defines the CAP rate. The absence of CAP for more than 60 s is scored as non-CAP (NCAP), and represents the portion of NREM sleep characterized by prolonged stationary activity. CAP sequences usually precede sleep stage transitions, and, specifically, subtypes A2 and A3 typically assist the shift from NREM to REM sleep. Under physiologic circumstances, CAP is not present during REM sleep. The following CAP variables were measured.

(i) Total CAP time in minutes (total CAP time in NREM sleep),
(ii) CAP rate (the ratio of CAP time over total NREM sleep time),
(iii) Number and duration of CAP cycles,
(iv) Number and duration of each phase A subtype (A1, A2, A3),
(v) Total number of phase A (derived by the sum of A1, A2, and A3),
(vi) Duration of phase A and B in seconds.

#### Neuronal avalanche analysis

Before performing avalanche analysis, waking and motion artifact segments during nocturnal sleep were manually identified and removed. Artifact-free EEG signals were *Z* score normalized to have zero mean and unit standard deviation (SD). To capture the spatiotemporal organization in avalanches of transient EEG events during sleep, we investigated clusters of large deflections of the artifact-free EEG signals. For each EEG channel, large positive or negative excursions beyond a threshold θ=± nSD were identified.

To define the threshold θ, we analyzed the distribution of EEG amplitudes (Figure 1B). A Gaussian distribution of amplitudes is expected to arise from a superposition of many uncorrelated sources. Conversely, EEG amplitude distributions deviate from a Gaussian shape, indicating presence of spatiotemporal correlations and collective behaviors involving different cortical areas (Figure 1). The comparison of the signal distribution to the best Gaussian fit indicates that the two distributions start to deviate from one another around θ=±2 SD (Figure 1). Thus, thresholds smaller than 2 SD would lead to the detection of many events related to noise in addition to real events whereas much larger thresholds will miss many of the real events. To avoid noise-related events while preserving most of relevant events, in this study we used a threshold value θ=±2 SD.

An avalanche was defined as a continuous time interval in which there is at least one excursion beyond threshold in at least one EEG channel (Figure 1). Avalanches are preceded and followed by time intervals with no excursions beyond threshold on any EEG channel.^30^^,^^54^ The size of an avalanche, *s*, was defined as the sum over all channels of the absolute values of the signals exceeding the threshold.

To characterize the relationship between the avalanche dynamics and the sleep macro-architecture, we calculate for each subject the avalanche density as a function of time, i.e., the fraction of time occupied by avalanches, measured as

(Equation 2) $$F_{av}\left(t\right)=\frac{u_{av}\left(t\right)}{u_{0}}$$

where $u_{av}\left(t\right)$ is the amount of time occupied by avalanches in a sliding window of length $T_{0}$ (sliding step = 1/(sampling frequency)), and $u_{0}=T_{0}$. The window length has been chosen equal to 10 s ($T_{0}=10$ s) as this is the order of magnitude of the largest avalanches in our recordings. In each sliding window of length $T_{0}$, we also evaluate the number of avalanches $N_{av}$ and the average duration ⟨T⟩ of all avalanches in that window.

To characterize the relationship between the avalanche dynamics and the sleep micro-architecture, we compute the Pearson correlation coefficient between the avalanche occurrence and the CAP measures, on a timescale dictated by the sampling rate of the recordings. Given the binary values $x_{i}=0,1$, $y_{i}=0,1$, where $x_{i}=1$ indicates the presence in the sample *i* of an ongoing avalanche, and $y_{i}=1$ indicates presence of a particular feature of the CAP framework (CAP, NCAP, subtypes A1, A2, A3, all A phases, phases B), we computes the Pearson correlation coefficient as:

(Equation 3) $$\rho_{x,y}=\frac{\sum_{i}^{N}\left(x_{i}-\bar{x}\right)\left(y_{i}-\bar{y}\right)}{\sqrt{\sum_{i}^{N}{\left(x_{i}-\bar{x}\right)}^{2}}\sqrt{\sum_{i}^{N}{\left(y_{i}-\bar{y}\right)}^{2}}}$$

where $\bar{x}=\sum_{i}^{N}x_{i}/N$ and *N* is the number of samples in the sleep recordings. Being binary values, the Pearson correlation coefficient is equivalent to the φ coefficient. The Pearson correlation coefficient has been also evaluated between avalanche occurrence and sleep stages using Equation 2, where $x_{i}=1$ indicates the presence ($x_{i}=0$ absence) in the sample *i* of an ongoing avalanche, and $y_{i}=1$ indicates presence ($y_{i}=0$ absence) of a particular sleep stage (REM,N1,N2,N3).

Although the thresholding procedure to define avalanches disregards smaller amplitude fluctuations and related contributions from fast oscillations, we notice that, in the context of our analysis, smaller amplitude fluctuations do not play a key role. Indeed, we show that that avalanche dynamics is stable over a wide range of thresholds, from 1 SD to 3 SD (Figure S3). Furthermore, the analysis prominently focuses on processes associated with slow oscillations, with strong predominance of slow-wave activity (delta oscillations, 0.5−4 Hz).

### Quantification and statistical analysis

Maximum likelihood estimation of power law exponents for avalanche size and duration distributions was performed using the Power law Python package.^61^ The power law fit minimized the Kolmogorov-Smirnov distance between original and fitted values, $D=sup_{x}\left|F_{data}\left(x\right)-F_{fit}\left(x\right)\right|$, where $F_{data}$ is the empirical cumulative distribution function (CDF) and $F_{fit}$ the fitted CDF. Power-law exponents for P(S) and P(T) were evaluated on pooled data in Figure 2, and the error on the fit plus a systematic error was reported. In all the other cases (Figure 3), the maximum likelihood estimates of the power law exponents τ and α were obtained for all individual subjects, and the mean with the corresponding standard error was used. The power law fit was compared to an exponential fit by evaluating the log likelihood ratio $R=\ln\;L_{p}/L_{e}$, where $L_{p,e}=\prod_{i=1}^{n}p_{p,e}\left(x_{i}\right)$ is the likelihood. *R* is positive if the data are more likely to follow a power law distribution, and negative if the data are more likely to follow exponential distribution. The statistical significance for *R* (p value) was estimated in the Power law Python package.^61^ For further details see.^100^

The systematic error (SE) on power law fit for the distribution P(s) and P(T) was estimated by varying the range of values—as specified by $x_{\min}$ and $x_{\max}$—used for the MLE of power law exponents on pooled data. Specifically, for P(s) we took $x_{\min}=0.5,1,2,3,4$ and 10 $x_{\max}$ values uniformly sampled between $x_{\max}=550$ and $x_{\max}=1000$, obtaining ⟨τ⟩=1.4669±0.0414. For P(T) we took $x_{\min}=0.025,0.03,0.035,0.04$ and 10 $x_{\max}$ values uniformly sampled between $x_{\max}=0.7$ and $x_{\max}=1.6$, obtaining ⟨α⟩=1.9809±0.0452. Thus, SE on τ and α is 0.0414 and 0.0452, respectively.

A one-way ANOVA test for group comparisons in case data passed the Shapiro-Wilk normality test was used; otherwise, Kruskal-Wallis one-way analysis of variance on ranks (ANOVA on ranks) was used. Multiple pairwise comparisons were performed with the Bonferroni correction. All statistical analyses were performed in MATLAB (Mathworks).

## Data Availability

• This study did not generate any new datasets. Data reported in this paper will be shared by the lead contact upon request. • Original codes used in this study are publicly available on GitHub (https://github.com/silvia1970/criticality). • Any additional information required to reanalyze the data reported in this paper is available from the lead contact upon request.
