# Supplementary material for: Criticality of neuronal avalanches in human sleep and their relationship with sleep macro- and micro-architecture
Source: iScience. 2023 Sep 9;26(10):107840. doi: 10.1016/j.isci.2023.107840 (PMC10520337; doi:10.1016/j.isci.2023.107840)
Supplement: Document S1. Figures S1–S8 and Table S1 [file mmc1.pdf]

iScience, Volume *26*

Supplemental information

Criticality of neuronal avalanches in human sleep and their relationship with sleep macro- and micro-architecture

Silvia Scarpetta, Niccolò Morisi, Carlotta Mutti, Nicoletta Azzi, Irene Trippi, Rosario Ciliento, Ilenia Apicella, Giovanni Messuti, Marianna Angiolelli, Fabrizio Lombardi, Liborio Parrino, and Anna Elisabetta Vaudano

Supplementary Information

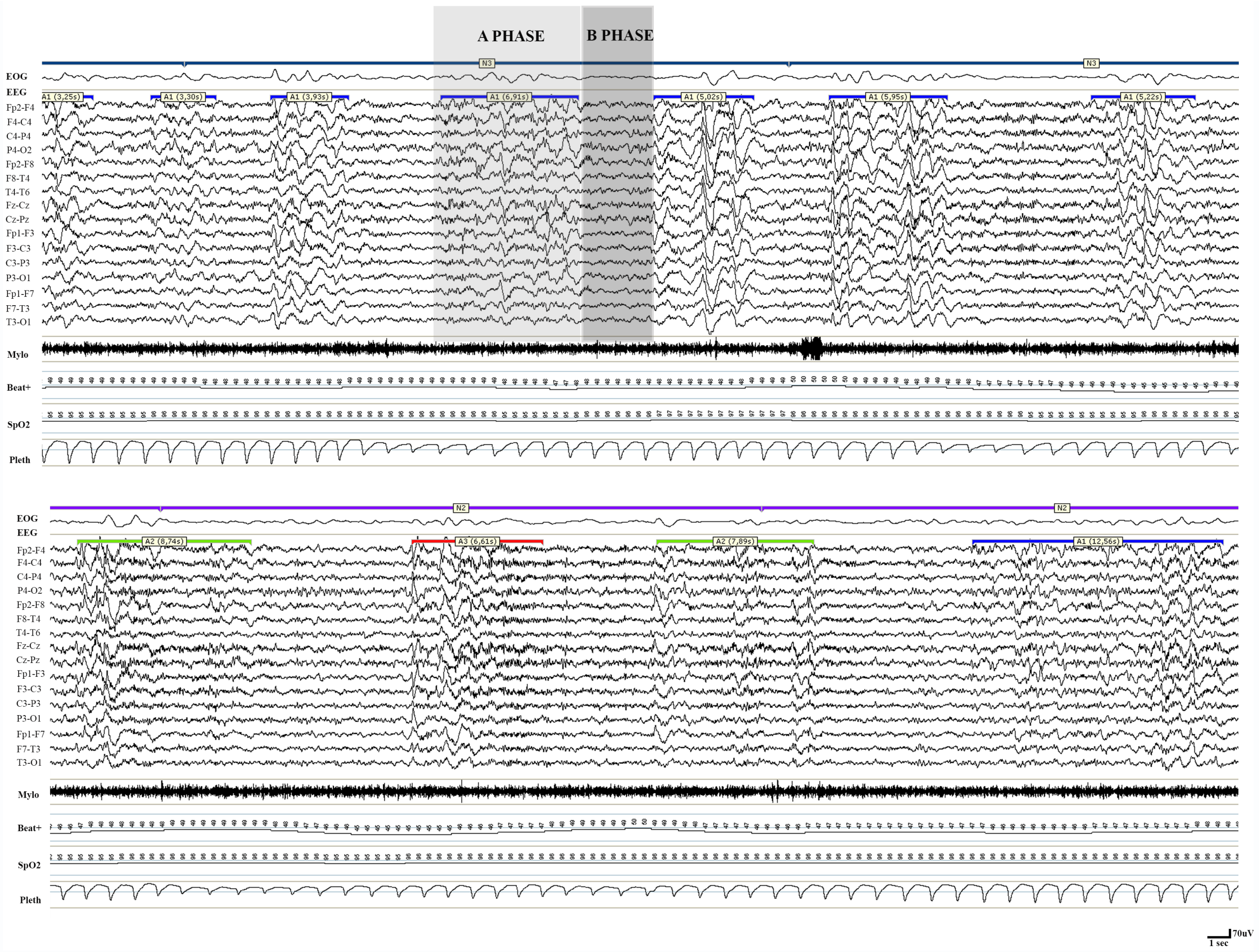

Fig. S1: **Examples of CAP during NREM sleep for an individual subject, related to Table 2. (A)** Two representative CAP during N3 NREM (top), N2 NREM (bottom). A and B phases are highlighted in the top panel (shaded areas) together with the A1 phases (blue lines). The bottom panel shows the subtypes A2 (green line) and A3 (red line). EEG (19 channels) is displayed in bipolar montage. EOG: Electro-oculogram. EMG derivation is represented by the activity of the mylohyoid muscle (mylo). Bpm: heart rate; SaO2: Oxyhaemoglobin saturation; Pleth: plethysmograph.

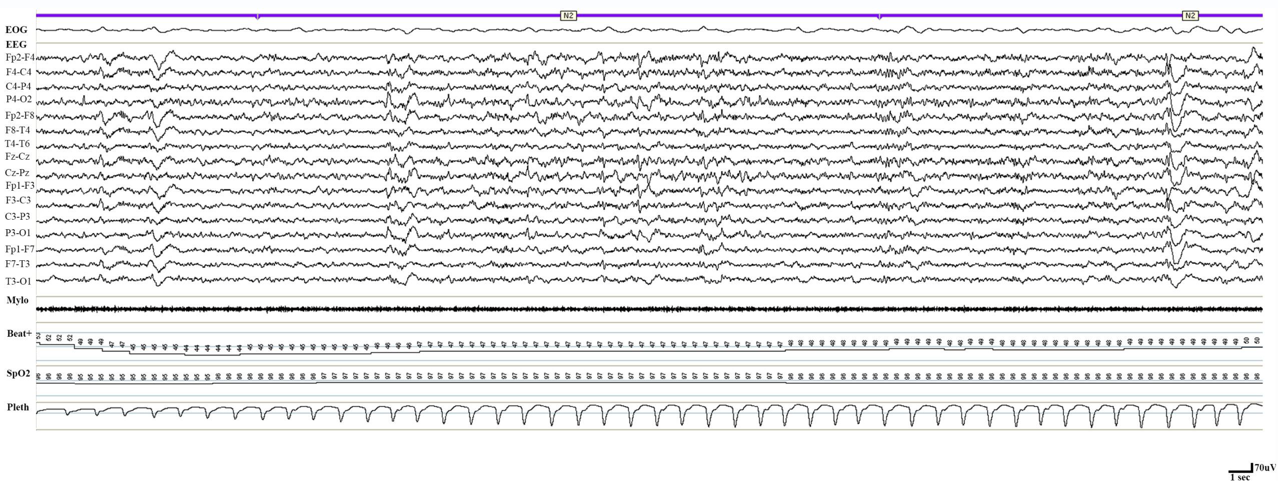

Fig. S2: **Examples of non-CAP (NCAP) during NREM sleep for an individual subject, related to Table 2.** NCAP during N2 NREM. EEG is displayed in bipolar montage. EOG: Electro-oculogram. EMG derivation is represented by the activity of the mylohyoid muscle (mylo). Bpm: heart rate; SaO2: Oxyhaemoglobin saturation; Pleth: plethysmograph.

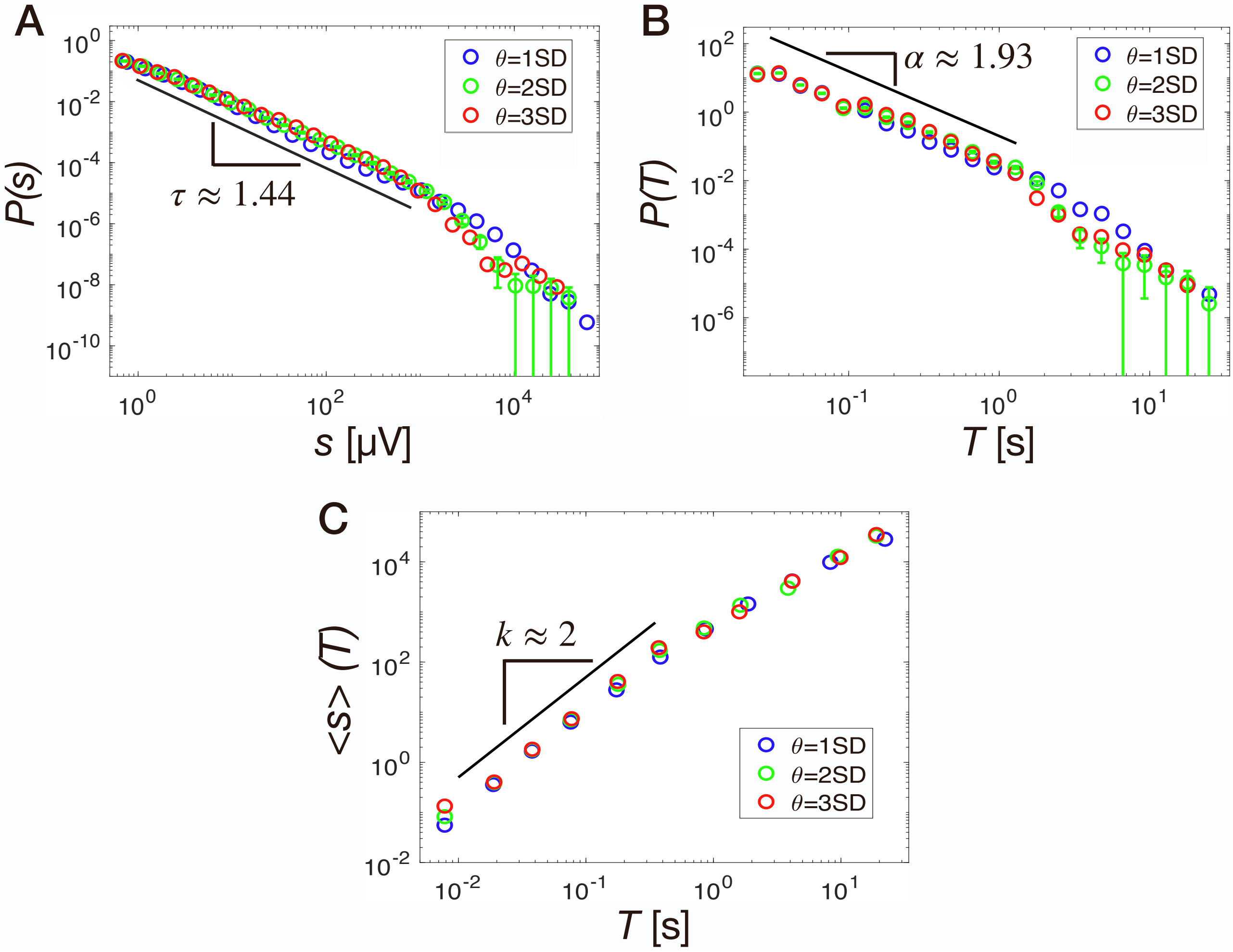

Fig. S3: **Avalanche dynamics weakly depends on the value of the threshold θ used to define the avalanches, related to STAR Methods.** Avalanche size (A) and duration distribution (B) for an individual subject and for different values of the threshold θ used to identify neuronal avalanches (STAR Methods) (blue circles: $\theta = 1$ SD; green circles: $\theta = 2$ SD; red circles: $\theta = 3$ SD). The error bars have been estimated assuming that the number of events in a particular interval $[x, x + dx]$ is given by the binomial distribution. This gives $\sigma_{P(x)} = P(x)\sqrt{(1 - n_x/N)/n_x}$, where n_x is the number of events in $[x, x + dx]$ and N is the total number of events. Error bars are shown only for threshold $\theta = 2$ SD, and represent two times $\sigma_{P(x)}$. The black line is the power law fit for $\theta = 2SD$. The average size as a function of the duration (C) follows the power law relationship $\langle s \rangle \propto T^k$ with $k = 2$ (black thick line) for all threshold values and for T's smaller than the onset of the cut-off regime of the duration distribution $P(T)$. For larger T's $k = 1.3$. The avalanche branching parameter [22] is always very close to 1 for all threshold values considered: $\theta = 1$ SD, $\sigma = 0.9999 \pm 0.0006$; $\theta = 2$ SD, $\sigma = 0.9989 \pm 0.0006$; $\theta = 3$ SD, $\sigma = 1.0000 \pm 0.0008$.

.

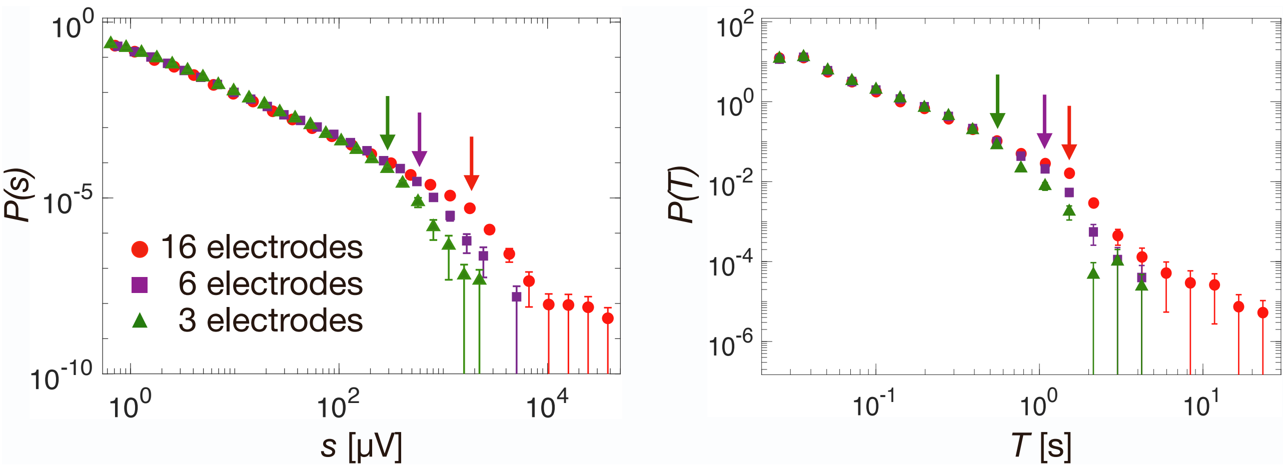

Fig. S4: **Analysis of finite size effects on the distributions of avalanche sizes and durations, related to Fig. 3.** Distributions of avalanche sizes (left) and durations (right) for different numbers of EEG electrodes (individual subject). Starting from the whole array (16 electrodes), we progressively removed electrodes from the analysis, moving from the right to the left hemisphere. Similar results were obtained when starting from the left hemisphere. Colored arrows indicate the onset of the cutoff on the corresponding distributions (red = 16, purple = 6, green = 3 electrodes). Error bars represent two times σ_P, calculated as in Fig. S3.

.

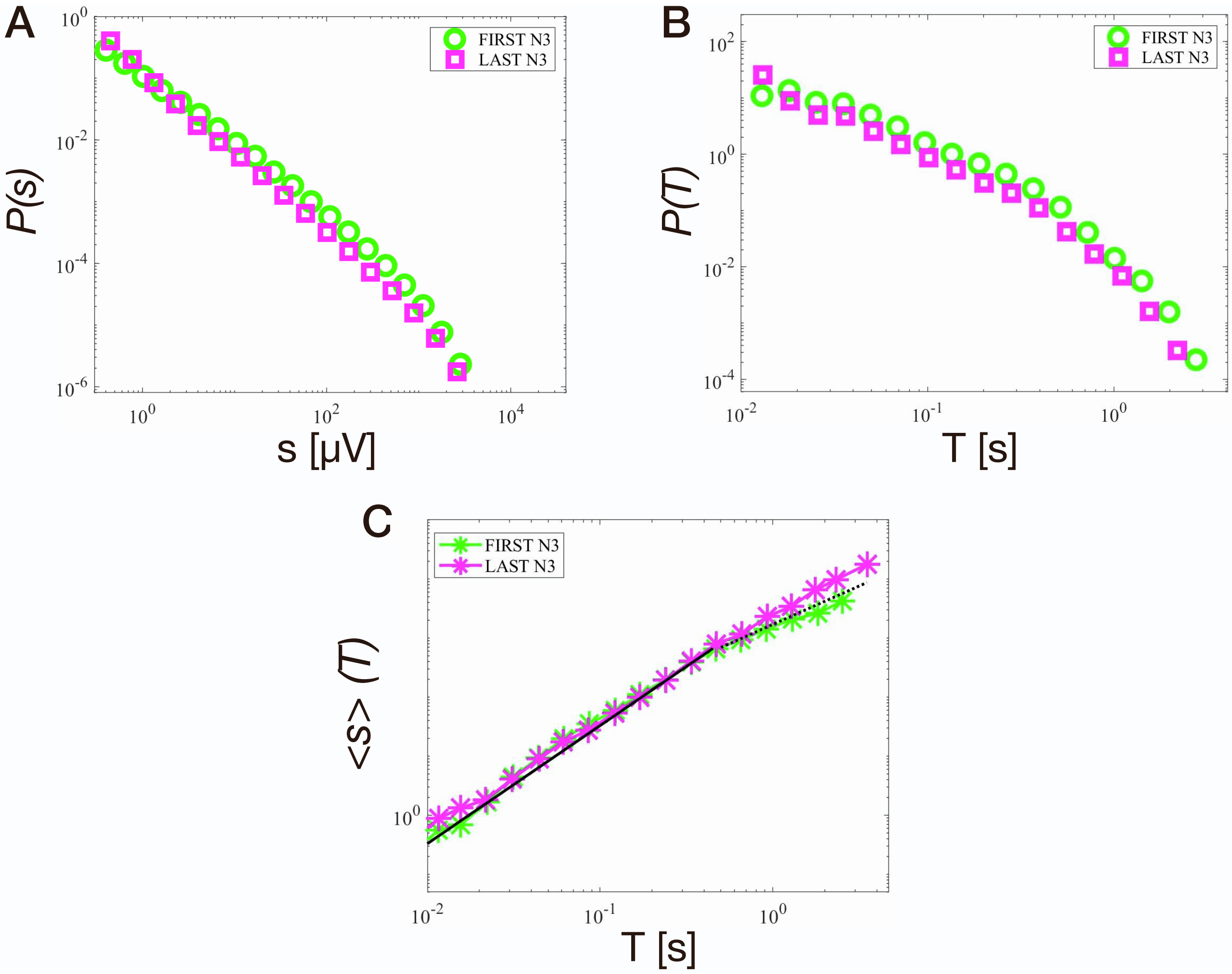

Fig. S5: **Avalanche dynamics remains stable during NREM sleep across the night, related to Fig.5.**. Distribution of size (A) and duration (B) for avalanches in the FIRST N3 (green) and the LAST N3 (magenta), and (C) average size as a function of the duration for avalanches in the FIRST N3 (green) and in the LAST N3 (magenta). Both the distributions and the relationship between average avalanche size and avalanche durations remain stable when moving from the FIRST N3 to the LAST N3.

Subj Id	TST (min)
1	427
2	453
3	451,5
4	435
5	340
6	295,5
7	529,5
8	433,5
9	493,5
10	380,5

Table S1: **Total sleep time for all individual subjects (n = 10), related to STAR Methods**. Total amount of sleep time scored during the total recording time for each subject (STAR Methods).

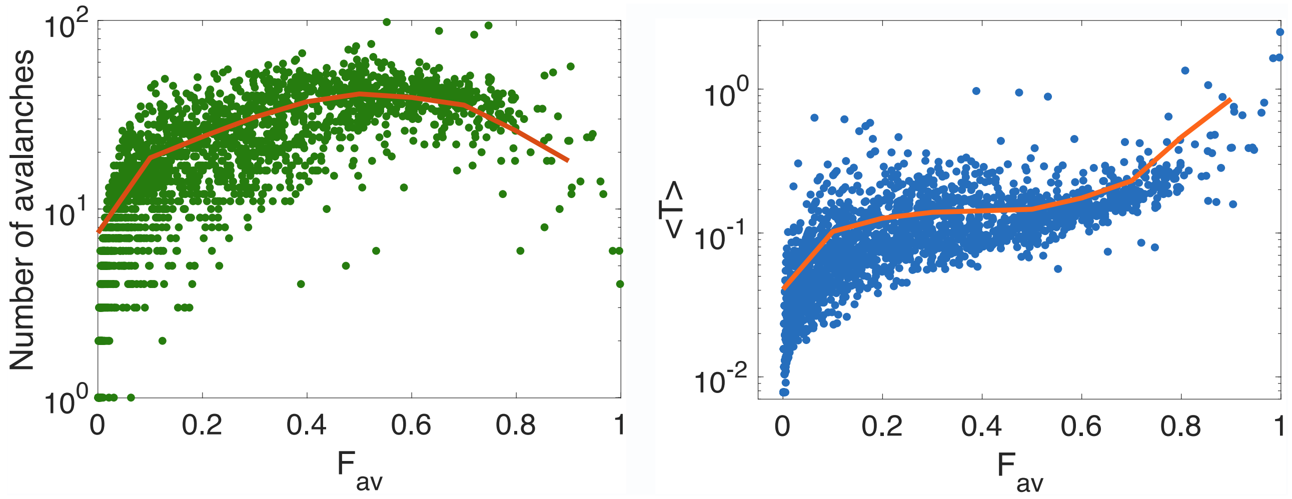

Fig. S6: **Relationship between the density of avalanches F_{av}, the number of avalanches, N_{av}, and the mean avalanche duration T in the sliding window T_0, related to Fig. 5.** Except for high densities F_{av}, the relationship between N_{av} and F_{av} (left), and T and F_{av} (right), is rather similar (both T and N_{av} tend to increase with F_{av}). On the other hand, relatively large densities ($F_av > 0.4$) do not correspond to a further increase in N_{av}, but are associated to occurrence of longer avalanches. In particular, T tends to increase for $F_{av} > 0.5$, while N_{av} tends to decrease. Data is shown for an individual subject.

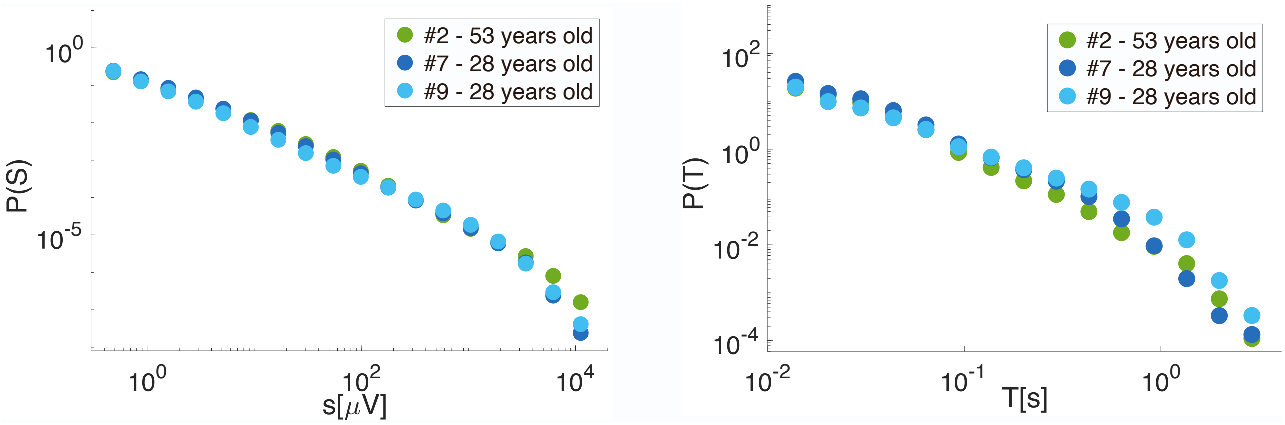

Fig. S7: **Avalanche size and duration distributions for the youngest and oldest subjects (28 vs 53 y), related to Fig. 3.** Both the size distribution (left) and the duration distribution (right) for the 53 y subject are very close to those from the 28 y subjects. Indeed, for both distributions, differences are no larger than those observed between the two youngest subjects (28 y).

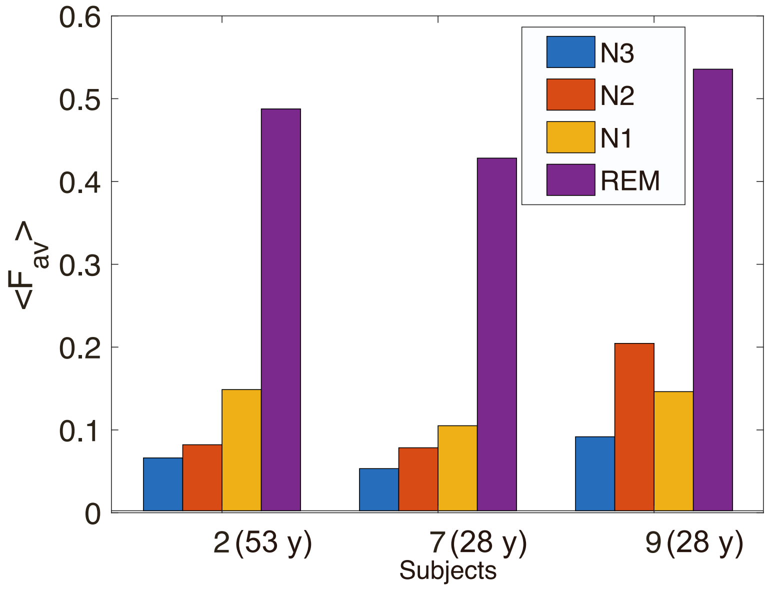

Fig. S8: **Avalanche density across sleep stages for the youngest and oldest subjects (28 vs 53 y), related to Fig. 5.** The oldest and youngest subject exhibit a similar pattern of avalanche density F_{av} across sleep stages.
